# SSN2: The next generation of spatial stream network modeling in R

**DOI:** 10.21105/joss.06389

**Published:** 2024-07-26

**Authors:** Michael Dumelle, Erin E. Peterson, Jay M. Ver Hoef, Alan Pearse, Daniel J. Isaak

**Affiliations:** 1 Pacific Ecological Systems Division, United States Environmental Protection Agency, Corvallis, OR, USA; 2 EP Consulting and Centre for Data Science, Queensland University of Technology, Brisbane, QLD, Australia; 3 NMFS Alaska Fisheries Science Center, United States National Oceanic and Atmospheric Administration, Seattle, WA, USA; 4 NIASRA, School of Mathematics and Applied Statistics, University of Wollongong, Wollongong, NSW, Australia; 5 Rocky Mountain Research Station, United States Forest Service, Boise, ID, USA

## Abstract

The SSN2 **R** package provides tools for spatial statistical modeling, parameter estimation, and prediction on stream (river) networks. SSN2 is the successor to the SSN **R** package (Ver Hoef, Peterson, Clifford, & Shah, 2014), which was archived alongside broader changes in the **R**-spatial ecosystem (Nowosad, 2023) that included 1) the retirement of rgdal (Bivand, Keitt, & Rowlingson, 2021), rgeos (Bivand & Rundel, 2020), and maptools (Bivand & Lewin-Koh, 2021) and 2) the lack of active development of sp (Bivand, Pebesma, & Gómez-Rubio, 2013). SSN2 maintains compatibility with the input data file structures used by the SSN **R** package but leverages modern **R**-spatial tools like sf (Pebesma, 2018). SSN2 also provides many useful features that were not available in the SSN **R** package, including new modeling and helper functions, enhanced fitting algorithms, and simplified syntax consistent with other **R** generic functions.

## Statement of Need

Streams provide vital aquatic services that sustain wildlife, provide drinking and irrigation water, and support recreational and cultural activities. Data are often collected at various locations on a stream network and used to characterize spatial patterns in stream phenomena. For example, a manager may need to know how the amount of a hazardous chemical changes throughout a stream network to inform mitigation efforts. Comprehensive formulations of spatial stream network (SSN) models are provided by Ver Hoef & Peterson (2010), Peterson & Ver Hoef (2010), and Ver Hoef et al. (2014). The SSN2 **R** package is designed to help users fit SSN models to their stream network data.

SSN models use a spatial statistical modeling framework (e.g., Cressie, 1993) to describe unique and complex dependencies on a stream network resulting from a branching network structure, directional water flow, and differences in flow volume. These SSN models relate a continuous or discrete response variable to one or more explanatory variables, a spatially independent random error term, and up to three spatially dependent random error terms: tail-up random errors, tail-down random errors, and Euclidean random errors. Tail-up random errors restrict spatial dependence to flow-connected sites (i.e., water flows from an upstream to a downstream site) and incorporate spatial weights through an additive function to describe the branching network between sites. Tail-down random errors describe spatial dependence between both flow-connected and flow-unconnected sites (i.e., sites that share a common downstream junction but not flow), but spatial weights are not required. Euclidean random errors describe spatial dependence between sites based on straight-line distance and are governed by factors not confined to the stream network, such as regional geology. The variances and the length-scales of spatial dependence in the tail-up, tail-down, and Euclidean random errors are controlled by separate variance (i.e., partial sill) and range parameters, respectively, while the spatially independent variance (i.e., nugget) is controlled by another separate variance parameter. In this paper, we show how to use the SSN2 **R** package to fit SSN models, inspect SSN models, and use SSN models to make predictions at unobserved locations on a stream network.

## Package Overview

The streams, observation, and prediction datasets must be pre-processed prior to fitting SSN models and making predictions at unobserved locations using SSN2. Previously, the STARS toolset for ArcGIS Desktop versions 9.3x - 10.8x (Peterson & Ver Hoef, 2014) or the openSTARS **R** package (Kattwinkel, Szöcs, Peterson, & Schäfer, 2020) were used to generate spatial information required for model fitting and prediction. However, both software packages have recently been retired and are replaced by the SSNbler **R** package (Peterson, Dumelle, Pearse, Teleki, & Ver Hoef, 2024), which is a new, **R**-based version of the STARS tools. SSNbler is currently available on GitHub, will soon be available on CRAN, and contains several useful resources that guide users through these pre-processing steps. Pre-processing using either SSNbler, STARS, or openSTARS ends with the creation of a .ssn folder, which is non-proprietary. Files residing in the .ssn folder are read into R using ssn_import() from SSN2 and placed into a list structure called an SSN object, which contains all the spatial, topological, and attribute information needed to leverage the modeling tools in SSN2.

SSN2 is first installed from CRAN:


install.packages(“SSN2”)


Then, SSN2 is loaded into an **R** session:


library(SSN2)


The SSN2 package comes with an example .ssn folder called MiddleFork04.ssn that represents water temperatures recorded from a stream network in the Middle Fork of the Salmon River in Idaho, USA during 2004.

Several functions in SSN2 for reading and writing data directly manipulate the .ssn folder. To avoid directly manipulating the MiddleFork04.ssn data installed alongside SSN2, MiddleFork04.ssn is instead copied into a temporary directory and the relevant path to this directory stored:


copy_lsn_to_temp()
path <- file.path(tempdir(), “MiddleFork04.ssn”)


**Figure 1:**
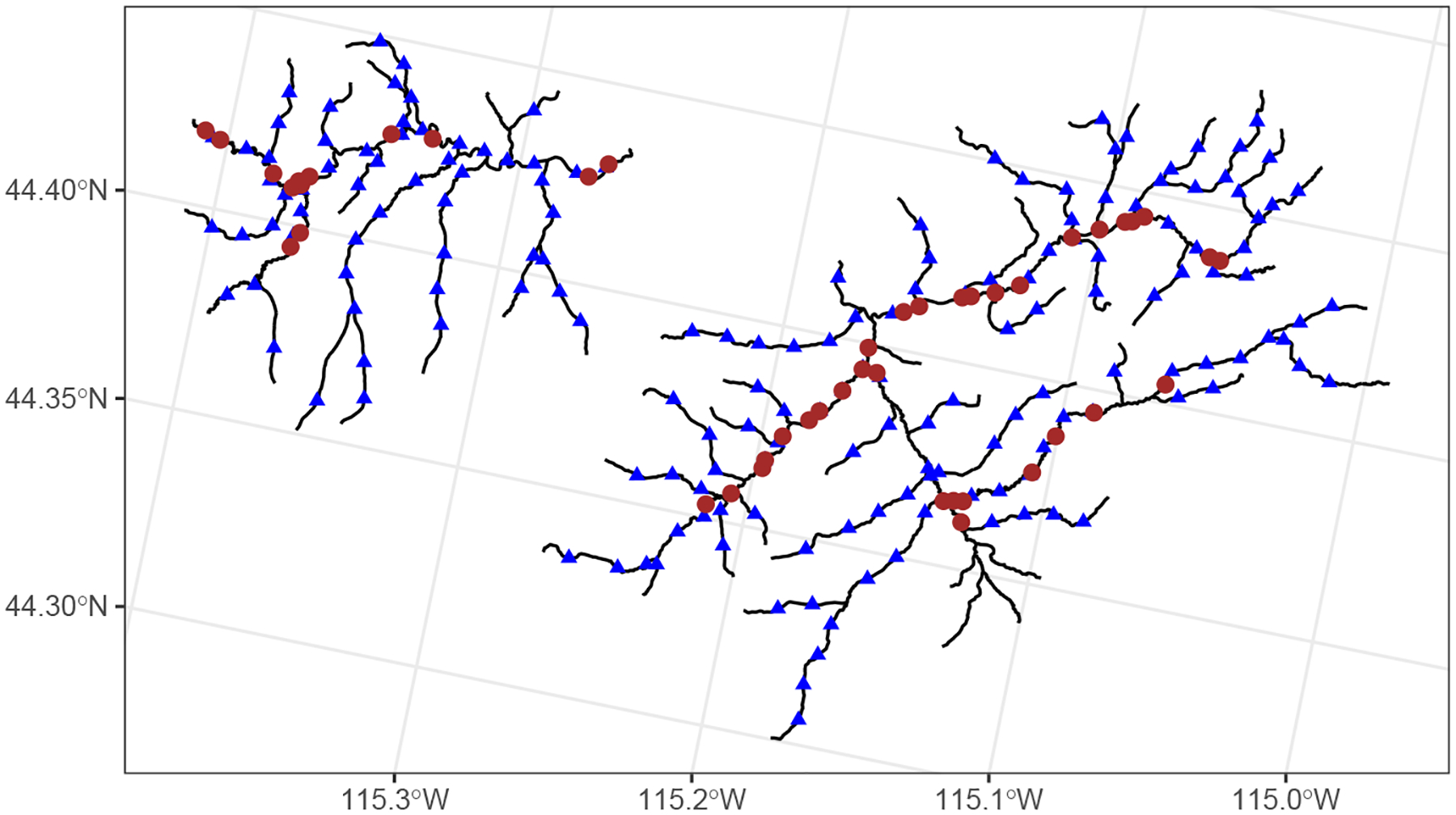
Middle Fork 2004 stream networks. Observed sites are represented by brown, closed circles at various locations throughout the stream network. Prediction sites are represented by blue, closed triangles and are spaced one kilometer apart.

The copy_lsn_to_temp() function is only used when working with MiddleFork04.ssn and generally, path should indicate a permanent directory on your computer that points towards your .ssn object. After specifying path, the stream reaches, observed sites, and prediction sites (pred1km) are imported and then visualized (Figure 1):


mf04p <- ssn_import(path, predpts = “pred1km”)
library(ggplot2)
ggplot() +
  geom_sf(data = mf04p$edges) +
  geom_sf(data = mf04p$preds$pred1km, pch = 17, color = “blue”) + 
  geom_sf(data = mf04p$obs, color = “brown”, size = 2) + 
  theme_bw()


Prior to statistical modeling, hydrologic distance matrices are created (Ver Hoef & Peterson, 2010):


ssn_create_distmat(mf04p, predpts = “pred1km”, overwrite = TRUE)


Of particular interest here is summer mean stream temperature (Summer_mn) in degrees Celsius, which will be modeled as a function of elevation (ELEV_DEM) and watershed-averaged precipitation (AREAWTMAP) with exponential, spherical, and Gaussian structures for the tail-up, tail-down, and Euclidean errors, respectively, and a nugget effect (by default). Using ssn_lm(), the model is fit:


ssn_mod <- ssn_lm(
  formula = Summer_mn ~ ELEV_DEM + AREAWTMAP, 
  ssn.object = mf04p,
  tailup_type = “exponential”, 
  taildown_type = “spherical”,
  euclid_type = “gaussian”,
  additive = “afvArea”
)


The additive argument represents an “additive function value (AFV)” variable that captures branching in the stream network and is required when modeling the tail-up covariance. Cumulative watershed area is commonly used to derive the additive function value (here, afvArea represents cumulative watershed area), but other variables like flow can be used (if every line feature in the edges dataset contains a non-null value). Ver Hoef & Peterson (2010) provide further details regarding additive function values.

The ssn_lm() function is designed to be similar in syntax and structure to the lm() function in base **R** for fitting nonspatial linear models. Additionally, SSN2 accommodates various S3 methods for commonly-used **R** generic functions that operate on model objects. For example, the generic function summary() is used to summarize the fitted model:


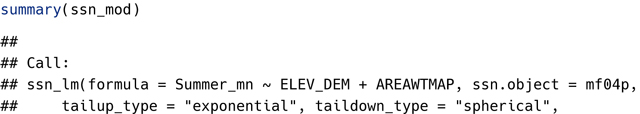



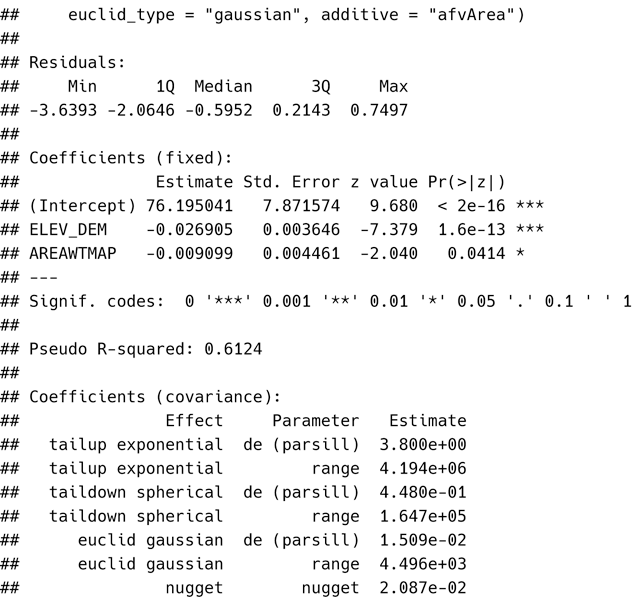


SSN2 methods for the tidy(), glance(), and augment() generic functions from the broom **R** package (Robinson, Hayes, & Couch, 2021) are used to inspect the fitted model and provide diagnostics:


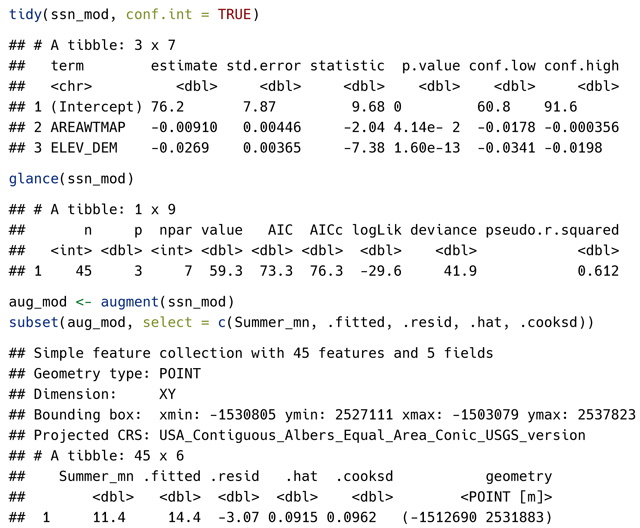



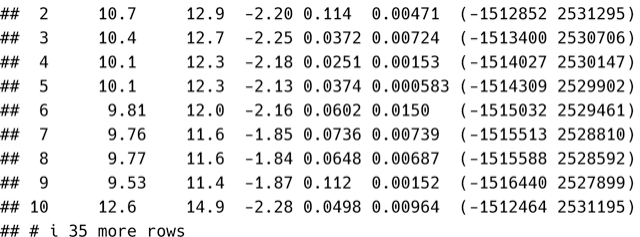


Specific generic helper functions (e.g., coef(), AIC(), residuals()) can be used to obtain the same quantities returned by tidy(), glance(), and augment():


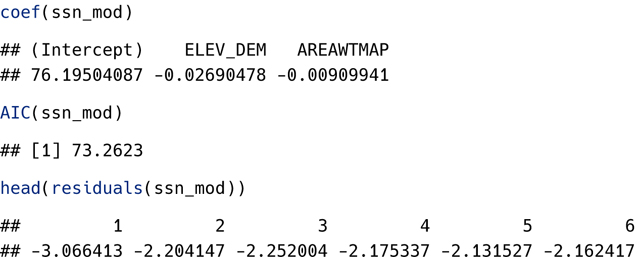


Spatial prediction (i.e., Kriging) at the unobserved sites is performed using the generic functions predict() or augment():


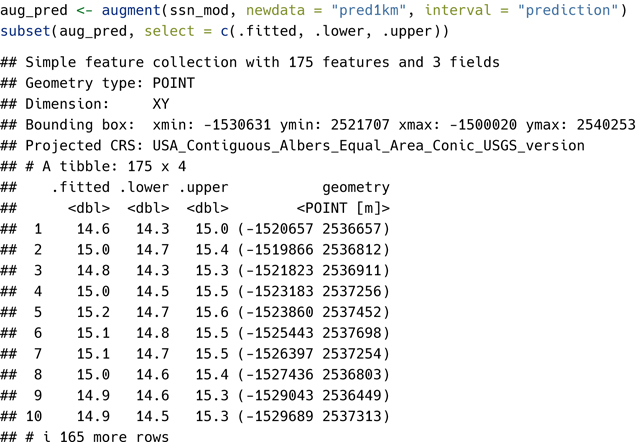


Here, .fitted are the predictions, .lower are the lower bounds of 95% prediction intervals, and .upper are the upper bounds of 95% prediction intervals. Utilizing augment() makes the prediction output straightforward to visualize:


ggplot() +
  geom_sf(data = mf04p$edges) +
  geom_sf(data = aug_pred, aes(color = .fitted), size = 2) +
  scale_color_viridis_c(name = “Pred.”, option = “H”) +
  theme_bw()


Spatial generalized linear models for binary, count, proportion, and skewed data (Ver Hoef et al., 2024) are applied to stream networks via the ssn_glm() function. ssn_lm() and ssn_glm() also accommodate several advanced features, which include nonspatial random effects as in lme4 (Bates, Mächler, Bolker, & Walker, 2015) and nlme (Pinheiro & Bates, 2006) Euclidean anisotropy (Zimmerman & Ver Hoef, 2024), and more. In addition to modeling, simulating data on a stream network is performed via ssn_simulate().

## Discussion

SSN models are valuable tools for statistical analysis of data collected on stream networks and help improve inference about vital stream ecosystems. These models have been employed to better understand and manage water quality (McManus et al., 2020; Scown, McManus, Carson Jr, & Nietch, 2017), ecosystem metabolism (Rodríguez-Castillo, Estévez, González-Ferreras, & Barquín, 2019), and climate change impacts on freshwater ecosystems (Isaak, Wenger, et al., 2017; Ruesch et al., 2012), as well as generate aquatic population estimates (Isaak, Ver Hoef, Peterson, Horan, & Nagel, 2017), inform conservation planning (Rodríguez-González et al., 2019; Sharma, Dubey, Johnson, Rawal, & Sivakumar, 2021), and assess restoration activities (Fuller, Leinenbach, Detenbeck, Labiosa, & Isaak, 2022), among other applications. The breadth and applicability of SSN models are further enhanced by data aggregation tools like the National Hydrography Dataset (McKay et al., 2012), National Stream Internet Project (Nagel, Peterson, Isaak, Ver Hoef, & Horan, 2015), and StreamCat (Hill, Weber, Leibowitz, Olsen, & Thornbrugh, 2016).

There are several spatial modeling packages in **R**, including geoR (Ribeiro Jr et al., 2022), gstat (Pebesma, 2004), FRK (Sainsbury-Dale, Zammit-Mangion, & Cressie, 2024), fields (Nychka, Furrer, Paige, & Sain, 2021), R-INLA (Lindgren & Rue, 2015), and spmodel (Dumelle, Higham, & Ver Hoef, 2023), among others. However, these aforementioned spatial modeling packages do not account for the unique spatial relationships found in data collected on stream networks. The rtop (Skoien et al., 2014), VAST (Charsley et al., 2023), and SSN2 **R** packages can be used to describe spatial stream network data in **R**, but SSN2 is unique. It not only provides representations of stream network data in **R** but also provides an extensive suite of functions for model fitting, diagnostics, and spatial prediction that integrate with the popular “tidy” framework (Kuhn & Silge, 2022; Wickham et al., 2019). To learn more about SSN2, visit the CRAN webpage at https://CRAN.R-project.org/package=SSN2.

## Figures and Tables

**Figure 2:**
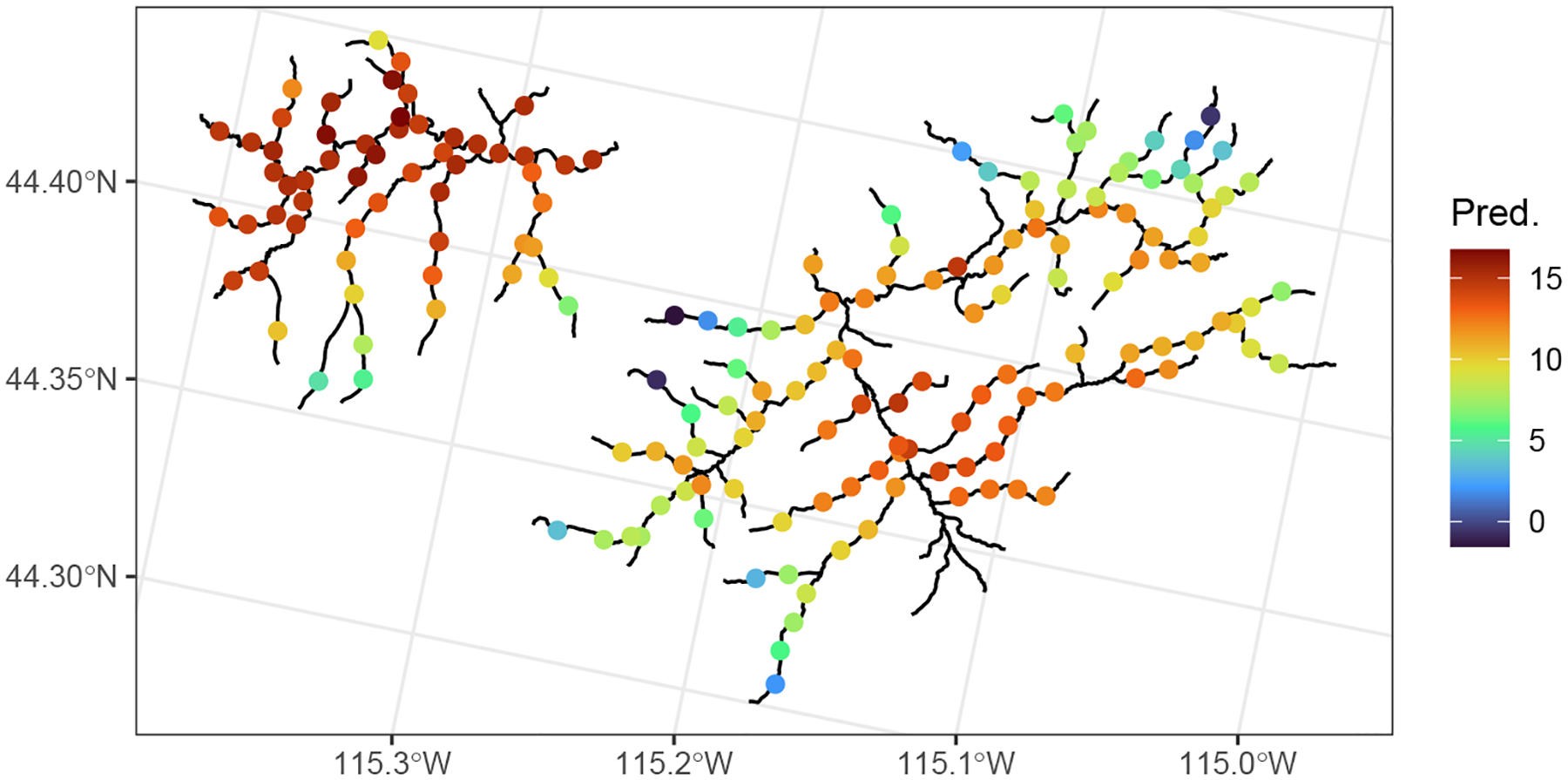
Predicted Middle Fork 2004 mean summer temperatures (Celsius) spaced one kilometer apart. As expected, temperature is predicted to be lower in areas of higher elevation.
